# Effect of Preoperative Risk Group Stratification on Oncologic Outcomes of Patients with Adverse Pathologic Findings at Radical Prostatectomy

**DOI:** 10.1371/journal.pone.0164497

**Published:** 2016-10-07

**Authors:** Won Sik Jang, Lawrence H. C. Kim, Cheol Yong Yoon, Koon Ho Rha, Young Deuk Choi, Sung Joon Hong, Won Sik Ham

**Affiliations:** Department of Urology, Urological Science Institute, Yonsei University College of Medicine, Seoul, Korea; National Health Research Institutes, TAIWAN

## Abstract

**Background:**

Current National Comprehensive Cancer Network guidelines recommend postoperative radiation therapy based only on adverse pathologic findings (APFs), irrespective of preoperative risk group. We assessed whether a model incorporating both the preoperative risk group and APFs could predict long-term oncologic outcomes better than a model based on APFs alone.

**Methods:**

We retrospectively reviewed 4,404 men who underwent radical prostatectomy (RP) at our institution between 1992 and 2014. After excluding patients receiving neoadjuvant therapy or with incomplete pathological or follow-up data, 3,092 men were included in the final analysis. APFs were defined as extraprostatic extension (EPE), seminal vesicle invasion (SVI), or a positive surgical margin (PSM). The adequacy of model fit to the data was compared using the likelihood-ratio test between the models with and without risk groups, and model discrimination was compared with the concordance index (c-index) for predicting biochemical recurrence (BCR) and prostate cancer-specific mortality (PCSM). We performed multivariate Cox proportional hazard model and competing risk regression analyses to identify predictors of BCR and PCSM in the total patient group and each of the risk groups.

**Results:**

Adding risk groups to the model containing only APFs significantly improved the fit to the data (likelihood-ratio test, p <0.001) and the c-index increased from 0.693 to 0.732 for BCR and from 0.707 to 0.747 for PCSM. A RP Gleason score (GS) ≥8 and a PSM were independently associated with BCR in the total patient group and also each risk group. However, only a GS ≥8 and SVI were associated with PCSM in the total patient group (GS ≥8: hazard ratio [HR] 5.39 and SVI: HR 3.36) and the high-risk group (GS ≥8: HR 6.31 and SVI: HR 4.05).

**Conclusion:**

The postoperative estimation of oncologic outcomes in men with APFs at RP was improved by considering preoperative risk group stratification. Although a PSM was an independent predictor for BCR, only a RP GS ≥8 and SVI were associated with PCSM in the total patient and high-risk groups.

## Introduction

Prostate cancer (PC) has become the most common non-dermatologic cancer among Western men.[1] Due to prostate-specific antigen (PSA) screening efforts, increasing numbers of PC cases are diagnosed when the tumor is still confined to the prostate.[2] The prolonged natural history of the disease as well as the potential risk of progression into metastasis and death must be taken into consideration during the initial management of newly-diagnosed PC. The initial evaluation, including serum PSA level, biopsy Gleason score (GS), and clinical T staging, determines risk stratification and assists in treatment decision-making. Several risk group stratifications, such as the D'Amico and National Comprehensive Cancer Network (NCCN), have been constructed to predict the oncologic outcome in patients with PC, and initial therapy for non-metastatic PC is determined according to these risk group stratifications.[3, 4]

Radical prostatectomy (RP) is one of the most commonly used treatments for patients with localized PC and a life expectancy of ≥10 years. However, approximately 30% of patients treated with RP have adverse pathologic findings (APFs).[5] Post-RP recurrence rates in patients with APFs may be greater than 60% at 5 years.[6] The American Urological Association (AUA) and American Society for Radiation Oncology (ASTRO) recommend that adjuvant radiotherapy (ART) should be offered for these patients with APFs because this therapy has been demonstrated to reduce biochemical recurrence (BCR), local recurrence, and clinical progression.[7] However, for the decision to administer ART is based only on the presence of APFs at RP, irrespective of preoperative risk group stratification.[8]

We hypothesized that the oncologic risk associated with APFs is highly influenced by the preoperative risk group of the patient. Therefore, we assessed whether a model incorporating the preoperative risk group and APFs can predict the long-term oncologic outcomes better than that based only on APFs.

## Materials and Methods

### Patient population

After Institutional Review Board approval was obtained, we performed a retrospective review of the data collected from our PC database on 4,404 patients who had undergone RP at our institution between 1992 and 2014. After exclusion of patients who received neoadjuvant therapy and those with incomplete pathological or follow-up data, 3,092 men were included in the final analysis. Patients with lymph node invasion at RP were excluded because they were candidates for adjuvant androgen deprivation therapy rather than ART.

### Patient characteristics

Clinical characteristics of the patients, including age, preoperative PSA level, clinical stage, and biopsy GS, were obtained through a review of our institutional medical records. TNM stage was determined according to the American Joint Committee on Cancer 7th edition TNM staging system.[9] All patients were stratified into low-, intermediate-, and high-risk groups according to the 2015 Prostate Cancer NCCN Guidelines Version 1.[4] APFs were defined as extraprostatic extension (EPE), seminal vesicle invasion (SVI), or a positive surgical margin (PSM).[7]

### Pathological analysis

As described previously, pathological analysis of RP specimens was performed by an experienced uropathologist at our institute.[10] Briefly, the entire surface of the resected prostate specimens was coated with India ink, fixed in neutral buffered formalin, and embedded in paraffin blocks. Whole-mount step sections were cut transversely at regular intervals from the apex of the prostate to the tips of the seminal vesicles. Each section was examined for SVI, EPE, and PSM.

### Follow-up

A postoperative PSA follow-up was undertaken at 3-month intervals for the first 2 years and at 6-month intervals for the next 3 years; an annual PSA follow-up was recommended thereafter. Administration of adjuvant or salvage radiotherapy (SRT) was at the discretion of the surgeon. BCR was defined as any two consecutive increases in serum PSA ≥0.2 ng/ml after RP. Biochemical recurrence–free survival (BCRFS) was defined as the time from the RP to the occurrence of BCR. Data regarding mortality and cause of death were obtained from medical records in the Cancer Registry Center database at our institution.[11] PC-specific mortality (PCSM) was designated when the underlying cause of death was PC or the patient had castration-resistant PC at the time of death.

### Statistical analysis

Baseline characteristics of men and pathologic outcomes were compared using χ2-tests for categorical data, and Student’s t-test or analysis of variance (ANOVA) test for continuous data. The adequacy of model fit to the data was compared between the models of APFs with and without risk groups using the likelihood-ratio test. Discrimination was compared using concordance index (c-index) for predicting BCR and PCSM.[12] The c-index is similar to an area under the receiver operating characteristic curve and is applicable to time-to-event data.[13] It values can range from 0.5, which indicates no predictive discrimination, to 1.0, which denotes a perfect separation of patients with different outcomes.[14] The Kaplan-Meier method with a log-rank test was used to estimate and compare the probabilities of BCR between groups. Cox proportional hazard models were used to investigate associations between variables and the risk of BCR. The cumulative incidence estimates of PCSM were compared between the groups using Gray's modified log-rank test for PCSM. To account for potential causes of death other than PC, a multivariate competing risk regression was also used to evaluate a possible association between the covariates and the risk of PCSM.[15] Covariates consisted of patient age (continuous), year of surgery (continuous), preoperative PSA level (continuous), RP GS (≤6, 7, and ≥8), RP T stage (OC, EPE, and SVI), PSM (categorical). Significant variables on univariate analysis were included in the multivariate model. The statistical analysis was conducted using Stata v.12.0 (StataCorp, College Station, TX, USA) and R (R version 3.0.1, R Foundation for Statistical Computing, Vienna, Austria). Comparisons with p values <0.05 were considered to be statistically significant.

## Results

### Descriptive statistics

Of 3,092 patients in the final cohort, 603 (19.5%), 1,031 (33.3%), and 1,458 (47.2%) patients were classified as low-risk, intermediate-risk, and high-risk groups, respectively, according to the NCCN risk stratification. Clinical and pathological features of all patients, stratified by preoperative risk group, are detailed in Table 1. There were significant differences in age, preoperative PSA level, biopsy GS, clinical stage, and APFs across the risk groups (p <0.001 for all).

**Table 1:** Comparison of clinical and pathological characteristics according to preoperative risk group.

		Preoperative risk group			
Variable	Total	Low	Intermediate	High	p value*
	3,092 (100)	603 (19.5)	1,031 (33.3)	1,458 (47.2)	
Age, years					<0.001**
Median	66	64	65	66	
IQR	61–70	59–69	61–70	62–71	
Year of surgery					0.050**
Median	2009	2009	2009	2010	
IQR	2007–2011	2007–2011	2007–2011	2008–2011	
PSA, ng/ml					<0.001**
Median	8.0	5.6	8.1	11.3	
IQR	5.3–13.9	4.4–7.0	5.3–12.0	6.5–23.4	
Biopsy GS					<0.001***
≤6	1,386 (44.8)	603 (100)	444 (43.1)	339 (23.3)	
7	980 (31.7)	0	587 (56.9)	393 (27.0)	
≥8	726 (23.5)	0	0	726 (49.7)	
Clinical T stage					<0.001***
≤T2	2,145 (69.4)	603 (100)	1031 (100)	555 (38.1)	
≥T3	947 (30.6)	0	0	903 (61.9)	
RP GS					<0.001***
≤6	865 (28.0)	371 (61.5)	297 (28.8)	197 (13.5)	
7	1,533 (49.6)	208 (34.5)	643 (62.4)	682 (46.8)	
≥8	694 (22.4)	24 (4.0)	91 (8.8)	579 (39.7)	
RP T stage					<0.001***
OC	1,343 (43.4)	380 (63.0)	505 (49.0)	458 (31.4)	
EPE	1,448 (46.8)	211 (35.0)	485 (47.0)	752 (51.6)	
SVI	301 (9.8)	12 (2.0)	41 (4.0)	248 (17.0)	
PSM					<0.001***
No	1,604 (51.9)	400 (66.3)	546 (53.0)	658 (45.1)	
Yes	1,488 (48.1)	203 (33.7)	485 (47.0)	800 (54.9)	

IQR = interquartile range; PSA = prostate-specific antigen; GS = Gleason score; RP = radical prostatectomy; OC = organ confined; EPE = extraprostatic extension; SVI = seminal vesicle invasion; PSM = positive surgical margin.

* p values are for comparison of low, intermediate, and high risk groups.

** p value derived from analysis of variance model.

*** p value derived from Pearson’s χ2-test.

### Comparison of the survival models’ performance

Table 2 shows comparison of the survival models’ performance between the two models of APFs with and without the preoperative risk group for BCR and PCSM. Adding preoperative risk groups to the model significantly improved the fit to the data for BCR and PCSM (likelihood-ratio test p <0.001 for both). The c-index also showed that the predictive value for BCR and PCSM increased considerably when the preoperative risk classification was incorporated into the model with APFs (c-index for BCR from 0.693 to 0.732 and for PCSM from 0.707 to 0.747).

**Table 2:** Comparison of the Survival Models’ Performance.

	Survival model	
	APFs	APFs + Preoperative risk group
c-index		
BCR	0.693	0.732
PCSM	0.707	0.747
Likelihood-ratio test		
BCR	p < 0.001	
PCSM	p < 0.001	

APF = adverse pathologic findings; c-index = concordance index; BCR = biochemical recurrence; PCSM = prostate cancer-specific mortality.

### Cox regression analysis of biochemical recurrence

**Fig 1:**
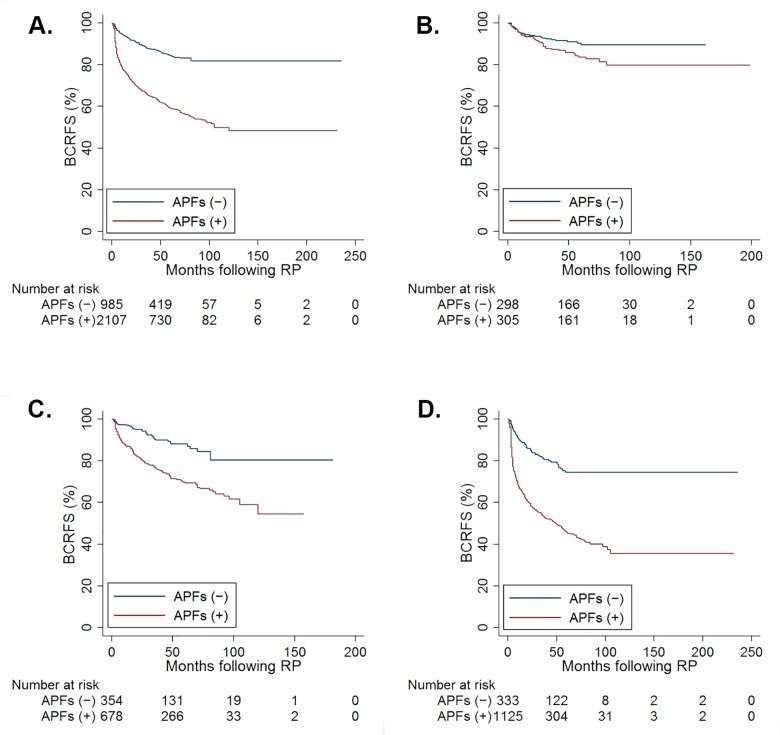
Kaplan-Meier estimates of biochemical recurrence–free survival (BCRFS) after radical prostatectomy (RP) by the presence of adverse pathological findings (APFs) for (A) total (log-rank test, p <0.001), (B) low-risk (p = 0.027), (C) intermediate-risk (p <0.001), and (D) high-risk (p <0.001) patients.

Of 3,092 patients, 899 men had experienced BCR at a median follow-up of 66 months. The BCRFS rate for men with APFs was worse than those without APFs in not only the total patient group but also in each risk group (total: p <0.001, low: p = 0.027, intermediate: p <0.001, and high: p <0.001; Fig 1A, 1B, 1C and 1D, respectively).

Table 3 shows the results of the multivariate Cox regression analyses predicting BCR following RP in all patients and the stratified cohort according to the preoperative risk group. For the total patient group, the year of surgery (hazard ratio [HR] 0.97, p <0.001), PSA (HR 1.00, p = 0.012), RP GS (GS 7: HR 2.18, p <0.001 vs. GS ≥8: HR 4.66, p <0.001), and APFs (EPE: HR 1.36, p <0.001; SVI: HR 2.45, p <0.001; and PSM: HR 1.93, p <0.001) were significantly associated with BCR. In the low-risk group, age (HR 1.04, p = 0.029), year of surgery (HR 0.85, p <0.001), RP GS ≥8 (HR 2.94, p = 0.007), and PSM (HR 1.87, p <0.010) were independent prognostic factors for BCR, while in the intermediate-risk group, BCR was significantly associated with PSA (HR 1.08, p <0.001), RP GS (GS 7: HR 1.73, p = 0.004 vs. GS ≥8: HR 1.85, p = 0.022), and APFs (SVI: HR 2.16, p = 0.002; PSM: HR 2.42, p <0.001). For the high-risk group, year of surgery (HR 0.98, p = 0.022), PSA (HR 1.00, p <0.001), RP GS (GS 7: HR 2.35, p <0.001 vs. GS ≥8: HR 4.63, p <0.001), and APFs (EPE: HR 1.51, p <0.001; SVI: HR 2.34, p <0.001; and PSM: HR 1.71, p <0.001) were significant predictors for BCR. EPE was not an independent prognostic factor of BCR for the low- and intermediate-risk groups (p = 0.910 and p = 0.923, respectively). There was also no association between SVI and BCR in the low-risk group (p = 0.118).

**Table 3:** Multivariate Cox regression analysis of biochemical recurrence according to preoperative risk group.

			Preoperative risk group					
Variable	Total		Low		Intermediate		High	
	HR (95% CI)	p value	HR (95% CI)	p value	HR (95% CI)	p value	HR (95% CI)	p value
Age, years	1.01 (0.99–1.02)	0.093	1.04 (1.00–1.08)	0.029				
Year of surgery	0.97 (0.95–0.98)	<0.001	0.85 (0.79–0.91)	<0.001	0.97 (0.94–1.02)	0.305	0.98 (0.95–0.99)	0.022
PSA, ng/ml	1.00 (1.00–1.00)	0.012	1.09 (0.96–1.24)	0.170	1.08 (1.05–1.11)	<0.001	1.00 (1.00–1.01)	<0.001
RP GS								
≤6	1 (Ref)		1 (Ref)		1 (Ref)		1 (Ref)	
7	2.18 (1.74–2.72)	<0.001	1.60 (0.97–2.68)	0.067	1.73 (1.20–2.50)	0.004	2.35 (1.59–3.48)	<0.001
≥8	4.66 (3.70–5.88)	<0.001	2.94 (1.34–6.48)	0.007	1.85 (1.09–3.12)	0.022	4.63 (3.15–6.82)	<0.001
RP T stage								
OC	1 (Ref)		1 (Ref)		1 (Ref)		1 (Ref)	
EPE	1.36 (1.14–1.61)	<0.001	0.97 (0.57–1.65)	0.910	1.02 (0.75–1.37)	0.923	1.51 (1.20–1.91)	<0.001
SVI	2.45 (1.97–3.04)	<0.001	2.23 (0.82–6.10)	0.118	2.16 (1.32–3.53)	0.002	2.34 (1.79–3.06)	<0.001
PSM								
No	1 (Ref)		1 (Ref)		1 (Ref)		1 (Ref)	
Yes	1.93 (1.66–2.24)	<0.001	1.87 (1.16–3.03)	0.010	2.42 (1.79–3.28)	<0.001	1.71 (1.42–2.06)	<0.001

PSA = prostate-specific antigen; RP = radical prostatectomy; GS = Gleason score; OC = organ confined; EPE = extraprostatic extension; SVI = seminal vesicle invasion; PSM = positive surgical margin; HR = hazard ratio; CI = confidence interval.

Significant variables on univariate analysis were included in the multivariate model.

### Competing risk regression analysis of prostate cancer-specific mortality

**Fig 2:**
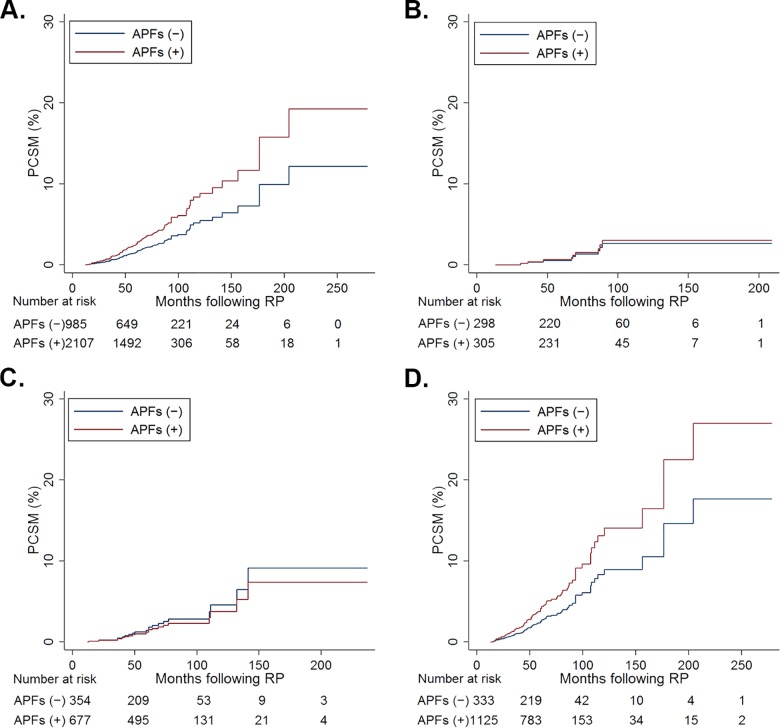
Cumulative incidence estimates of prostate cancer-specific mortality (PCSM) after radical prostatectomy (RP) using a competing risk analysis by adverse pathological findings (APFs) for (A) total (Gray’s modified log rank, p = 0.001), (B) low-risk (p = 0.903), (C) intermediate-risk (p = 0.253), and (D) high-risk (p = 0.010) patients.

Of 3,092 patients, 85 men (low: 8, intermediate: 13, and high: 63) died due to PC. The cumulative incidence estimates of PCSM for men with APFs was higher than those without APFs in not only the total patient group but also the high-risk group (Gray’s modified log rank, p = 0.001 and p = 0.010, respectively), while the estimate was not elevated in the low- and intermediate-risk groups (p = 0.903 and p = 0.253, respectively; Fig 2).

In the multivariate competing risk regression analysis, RP GS ≥8 and SVI were independent predictors for PCSM in the total patient group (GS ≥8: HR 5.50, p <0.001; SVI: HR 3.02, p = 0.002) and the high-risk group (GS ≥8: HR 6.21, p = 0.011; SVI: HR 3.46, p = 0.005), while EPE and PSM were not (Table 4).

**Table 4:** Multivariate competing risk regression analysis of prostate cancer-specific mortality in overall patients and the high-risk group.

Variable	Total		High risk	
	HR (95% CI)	p value	HR (95% CI)	p value
Age, years	1.03 (0.99–1.07)	0.053	1.04 (0.99–1.09)	0.082
Year of surgery	0.94 (0.87–1.01)	0.073		
PSA, ng/ml	1.00 (0.99–1.00)	0.633		
RP GS				
≤6	1 (Ref)		1 (Ref)	
7	1.34 (0.61–2.94)	0.466	1.88 (0.43–8.11)	0.398
≥8	5.50 (2.62–11.56)	<0.001	6.21 (1.52–25.3)	0.011
RP T stage				
OC	1 (Ref)		1 (Ref)	
EPE	1.41 (0.77–2.58)	0.270	1.76 (0.77–4.03)	0.181
SVI	3.02 (1.50–6.06)	0.002	3.46 (1.46–8.24)	0.005
PSM				
No	1 (Ref)		1 (Ref)	
Yes	1.09 (0.67–1.76)	0.729	1.35 (0.75–2.41)	0.319

PSA = prostate-specific antigen; RP = radical prostatectomy; GS = Gleason score; OC = organ confined; EPE = extraprostatic extension; SVI = seminal vesicle invasion; HR = hazard ratio; CI = confidence interval.

Significant variables on univariate analysis were included in the multivariate model.

## Discussion

Approximately 60% of patients with APFs after RP will experience BCR.[6] In this context, the AUA/ASTRO guideline recommends ART to patients with APF at RP and SRT to patients with biochemical or local recurrence after RP in whom there is no evidence of distant metastatic disease through an in-depth discussion of possible side effects of radiotherapy as well as the potential benefits of preventing recurrence.[7] The AUA/ASTRO guideline for ART is largely based on three randomized clinical trials (SWOG 8794, EORTC 22911, and ARO 96–02).[16–18] These randomized clinical trials have shown that ART after RP reduces the risk of BCR for patients with APFs. In addition, SWOG 8794 reported improved overall survival with ART compared to observation alone.

Even though this guideline is in place, clinicians differ in their opinions and practice with regards to the provision of ART.[19] This variability is partly due to a risk of functional complications such as incontinence and impotence while the oncological benefit may not be clinically significant.[20] The SWOG 8794 trial reported that patients who underwent SRT after BCR had a similar overall survival rate compared to those who underwent ART with an undetectable PSA level after RP.[21] Soloway et al. reported that patients with a PSM who underwent ART and recurred had similar long-term outcomes compared to those who underwent SRT after BCR.[22] These results suggest that ART is not necessary for all patients with APFs after RP. Kang et al. subsequently evaluated patients with APFs who meet the current AUA/ASTRO guideline for ART. They found that only 16.6% of ART patients developed BCR. In addition, in 87 patients with a preoperative PSA <6.35 ng/ml and a GS <8, only three were recurred (3.4%). Thus, they recommended a more customized approach to selecting patients for ART to avoid significant overtreatment.[23] Swanson et al. also concluded that the risk of BCR in men with locally advanced disease varies widely depending on the preoperative PSA value (<10 vs. ≥10 ng/ml) and RP GS (<7 vs. ≥7).[5]

NCCN preoperative risk group stratification has been widely adopted as a mainstay of treatment criteria prior to making a definitive treatment. Extending its usefulness and the significance of preoperative risk group stratification to post-RP patients with APFs would be reasonable. Based on this hypothesis, we believed that preoperative risk group stratification may also influence oncologic risk associated with APFs and could play an adjunctive role in the selection of optimal candidates for ART after RP. Therefore, we assessed whether a model incorporating preoperative risk and APFs could predict long-term oncologic outcomes better than that based only on APFs.

Imnadze et al. recently reported that the risk of BCR in men with APFs is dramatically attenuated by a low preoperative risk status, which reduces the risk associated with findings such as ECE or high (>50%) Gleason grade disease. This finding suggests that preoperative risk group stratification is an important factor to consider when evaluating the post-RP risk of BCR in the context of APFs.[8] However, this study was limited by its use of only BCR as an end-point.

In our present study, we showed the additive prognostic value of preoperative risk group stratification for postoperative oncologic outcomes in patients with APFs. We demonstrated that APFs at RP are associated with an increased risk of BCR and PCSM, but this oncologic risk is highly influenced by preoperative risk group. A PSM, which has been a common indication for ART, was independently associated with BCR in not only the total patient group but also each of the risk groups, while RP GS ≥8 and SVI were associated with PCSM in the total patient and the high-risk groups. These findings suggest that additional stratification by preoperative risk group can more accurately predict oncological outcomes of patients with APFs.

This study has several limitations. First, all data were reviewed retrospectively from a single institution; therefore, our results may not be generalizable. Second, information about adjuvant or salvage therapy was not included in the analysis because only a few men received ART and because salvage therapy can act as a surrogate marker of BCR.[24, 25] Finally, a major limitation was that we did not perform a competing risk analysis for PCSM in the low- and intermediate-risk groups because of the small number of events. To better assess the effect of preoperative risk group on these groups, a larger sample size and longer follow-up will be required.

## Conclusions

Our results show that the postoperative estimation of oncologic outcomes in men with APFs at RP is improved by considering preoperative risk group stratification. Although a PSM was an independent predictor for BCR, only an RP GS ≥8 and SVI were associated with PCSM in the total patient and high-risk groups. These findings suggest that preoperative risk group stratification should be considered in the selection of optimal ART candidates after RP, although our present results need to be validated by future studies before making a recommendation.

## Supporting Information

S1 File Raw data of study cohort. (XLS)
